# *T*_2_ mapping of cerebrospinal fluid: 3 T versus 7 T

**DOI:** 10.1007/s10334-017-0659-3

**Published:** 2017-11-06

**Authors:** Jolanda M. Spijkerman, Esben T. Petersen, Jeroen Hendrikse, Peter Luijten, Jaco J. M. Zwanenburg

**Affiliations:** 1 Department of Radiology, University Medical Center Utrecht, HP E 01.132, P.O.Box 85500, 3508 GA Utrecht, The Netherlands; 2 Danish Research Centre for Magnetic Resonance, Centre for Functional and Diagnostic Imaging and Research, Copenhagen University Hospital Hvidovre, 2650 Hvidovre, Denmark

**Keywords:** Cerebrospinal fluid, T2 relaxation, 7 T, 3 T, MRI

## Abstract

**Object:**

Cerebrospinal fluid (CSF) *T*_2_ mapping can potentially be used to investigate CSF composition. A previously proposed CSF *T*_2_–mapping method reported a *T*_2_ difference between peripheral and ventricular CSF, and suggested that this reflected different CSF compositions. We studied the performance of this method at 7 T and evaluated the influence of partial volume and ***B***_1_ and ***B***_0_ inhomogeneity.

**Materials and methods:**

*T*_2_-preparation-based CSF *T*_2_-mapping was performed in seven healthy volunteers at 7 and 3 T, and was compared with a single echo spin-echo sequence with various echo times. The influence of partial volume was assessed by our analyzing the longest echo times only. ***B***_1_ and ***B***_0_ maps were acquired. ***B***_1_ and ***B***_0_ dependency of the sequences was tested with a phantom.

**Results:**

*T*_2,CSF_ was shorter at 7 T compared with 3 T. At 3 T, but not at 7 T, peripheral *T*_2,CSF_ was significantly shorter than ventricular *T*_2,CSF_. Partial volume contributed to this *T*_2_ difference, but could not fully explain it. ***B***_1_ and ***B***_0_ inhomogeneity had only a very limited effect. *T*_2,CSF_ did not depend on the voxel size, probably because of the used method to select of the regions of interest.

**Conclusion:**

CSF *T*_2_ mapping is feasible at 7 T. The shorter peripheral *T*_2,CSF_ is likely a combined effect of partial volume and CSF composition.

**Electronic supplementary material:**

The online version of this article (doi:10.1007/s10334-017-0659-3) contains supplementary material, which is available to authorized users.

## Introduction

Qin [1] proposed a fast 3-T MRI method to map the volume and *T*_2_ of cerebrospinal fluid (CSF) in the brain. A striking finding with this method was the observation of a shorter *T*_2_ in the peripheral CSF compared with the *T*_2_ of the CSF in the lateral ventricles. Qin suggested that this *T*_2_ difference is caused by differences in CSF composition between both areas, implying that CSF *T*_2_ (*T*_2,CSF_) can be used as a noninvasive biomarker for CSF composition. This would be highly relevant in the light of the recent attention to the clearance of brain waste products, in which CSF is involved [2–5]. A method that can noninvasively assess CSF composition would provide a noninvasive window on the brain clearance system, with great potential for applications in studying diseases related to dementia such as Alzheimer’s disease and cerebral small vessel disease. If *T*_2,CSF_ is indeed useful as a functional marker of the brain clearance system, it could be studied next to other advanced imaging markers of early brain damage such as microbleeds, microinfarcts, and hippocampus subfield volumes and atrophy. As many of these advanced markers are acquired at 7 T [6–8], it is desirable to implement and evaluate CSF *T*_2_ mapping at 7 T as well.

At 7 T, ***B***_1_ inhomogeneity is considerable and may influence the *T*_2_ mapping results, despite the relative ***B***_1_ insensitivity of the used CSF *T*_2_ mapping method. Even at 3 T, considerable ***B***_1_ inhomogeneity in the brain can be observed [9]. Also, when *T*_2_ is measured in peripheral CSF, partial volume effects with tissue cannot be avoided. So, we hypothesized that these partial volume effects and ***B***_1_ imperfections can explain the previously observed *T*_2_ differences. De Vis et al. [10] obtained a rough estimation of the influence of partial volume effects on the estimated *T*_2,CSF_ by scanning with two different resolutions. The higher resolution resulted in longer *T*_2_ times, suggesting a role for partial volume effects. Because the influence of ***B***_1_ inhomogeneity and partial volume effects is not clear yet, it remains uncertain to what extent *T*_2,CSF_ can be used to assess the composition of CSF.

In this work we studied the performance of Qin’s CSF *T*_2_ mapping method at 7 T. The specific goals were to investigate the influence of ***B***_1_ and ***B***_0_ imperfections on the estimated *T*_2,CSF_, to assess the influence of partial volume effects, and to evaluate to what extent the previously observed difference in *T*_2,CSF_ between periphery and ventricles can be explained by ***B***_1_, ***B***_0_, and partial volume effects. ***B***_1_ and ***B***_0_ sensitivity was investigated with phantom measurements, and by comparison of the method for 7 and 3 T in healthy humans. Partial volume effects were estimated by removal of the influence of partial volume with tissue, through selection of only the last (longest) echo times (TEs). Also, scanning was done at different resolutions.

## Materials and methods

### Sequence

**Fig. 1:**
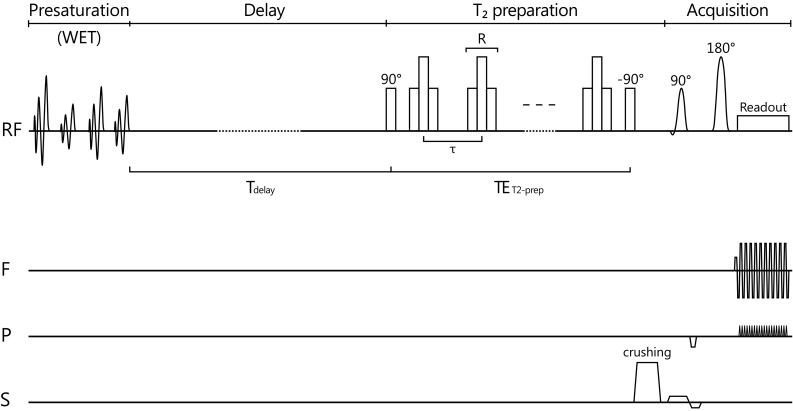
Cerebrospinal fluid *T*_2_ mapping pulse sequence. The sequence consists of four parts: water suppression enhanced through T_1_ effects (WET) presaturation, a fixed delay with duration *T*_delay_, a *T*_2_ preparation module with Malcolm Levitt (MLEV) phase cycling, and a spin echo echo planar imaging (SE-EPI) image acquisition. T_2_ relaxation occurs during TE_T2-prep_. To perform *T*_2_ mapping the sequence was repeated, while the number of refocusing pulses (and therefore TE_T2-prep_) was increased for a fixed interpulse delay *τ*. The applied RF pulses are shown on the RF axis, and the applied gradients are shown on the frequency (F), phase (P), and slice (S) encoding axes

The CSF *T*_2_ mapping sequence used in this research is based on *T*_2_ preparation, and has been described elsewhere [1, 10]. For this study the method was further extended to improve the fit reliability of the long *T*_2_ times by implementation of longer refocusing pulse trains, yielding longer TEs. Briefly, the sequence consists of four parts (Fig. 1). First, a set of four nonselective water suppression enhanced through *T*_1_ effects (WET) pulses are applied for saturation to prevent slice history effects. These pulses are optimized for saturation of free water (applicable for *T*_1_ between 3 and 6 s); the pulse angles are 156°, 71°, 109°, and 90° [11]. Second, a delay time (*T*_delay_) follows, where *T*_1_ relaxation occurs, followed by crusher gradients. Third, *T*_2_ preparation is applied, consisting of a nonselective 90° pulse, a set of 4, 8, 16, or 32 nonselective refocusing pulses (*R*) according to the Malcolm Levitt (MLEV) phase cycling scheme, and a nonselective −90° pulse with a crusher gradient to crush any remaining transverse magnetization [12]. Each refocusing pulse *R* is a composite pulse, consisting of $90_{\text{x}}^{ \circ } , \, 180_{\text{y}}^{ \circ } , \, 90_{\text{x}}^{ \circ }$ rectangular pulses (or the inverse ${\bar{\text{R}}}:90_{\text{ -x}}^{ \circ } , \, 180_{\text{ -y}}^{ \circ } , \, 90_{\text{ -x}}^{ \circ }$). The duration of a single refocusing pulse was 2.6 ms. *T*_2_ relaxation occurs during TE_T2-prep_, which is determined by the number of refocusing pulses and the spacing between the centers of the refocusing pulses (*τ*). To achieve long TE_T2-prep_ times, *τ* was chosen as 150 ms. This resulted in TE_T2-prep_ durations of 600, 1200, 2400, and 4800 ms. Also, one scan was acquired without any refocusing pulses; this scan was not used in data analysis. The fourth part of the sequence is a single-shot 2D spin echo (SE) echo planar imaging (EPI) readout. During the EPI train, *T*_2_ decay also occurs; this can, however, be regarded as a constant factor, and was therefore disregarded in the analysis.

Although the refocusing train in the *T*_2_ preparation is relatively insensitive to ***B***_1_ inhomogeneities because of the MLEV phase cycling scheme, the 90° rectangular pulses before and after the train may fail in the case of ***B***_1_ deviations. Consequently, we hypothesized that a fraction of the magnetization may be unaffected by the *T*_2_-preparation module, which could have a relatively large impact on the measured signal in the case of partial volume effects. Also, in the case of imperfect ***B***_1_, *T*_1_-weighted stimulated echoes may influence the *T*_2_ measurements, although this effect is expected to be relatively small because of the long *T*_1_ of CSF (4.4 s [13]). Therefore, *T*_2_ mapping with a single echo SE-EPI sequence with various TEs was used as a truly ***B***_1_-insensitive reference (shown in the electronic supplemental material).

Nonselective pulses were used where possible to minimize motion sensitivity. Consequently, only the excitation pulse of the SE-EPI readout was selective.

### Phantom measurements

Phantom measurements were performed to test the ***B***_1_ and ***B***_0_ sensitivity. The experiments were performed with a 7-T Philips Achieva scanner (Philips Medical Systems, Best, Netherlands) with a 32-channel head coil (Nova Medical, Wilmington, MA, USA), with a tap water phantom at room temperature. The phantom size was 200 × 95 × 20 mm^3^. The phantom was scanned with the same TEs as used for the in vivo experiments. A single slice was acquired with 3 × 3 × 6 mm^3^ resolution, field of view (FOV) of 240 × 96 mm^2^, *T*_delay_ of 15 s, [which is more than three times the *T*_1_ of CSF (4.4 s [13])], and sensitivity encoding (SENSE) [14] (with SENSE factor 1, meaning that the coil sensitivities of the receive coils were used for optimal coil combination without imaging acceleration). A series of *T*_2_ maps with increasing through-plane ***B***_0_ gradients were acquired to study the effect of diffusion for ***B***_1_ of 100%. The following through-plane ***B***_0_ gradient strengths were applied by addition of this strength to the linear shim term in the user interface: 0, 0.05, 0.1, 0.2, 0.3, and 0.5 mT/m. The phantom appeared sensitive to free induction decay (FID) artifacts, because of the relatively large volume of water and more pronounced ***B***_1_ inhomogeneity [15]. The acquisition with the highest ***B***_0_ gradient yielded images free of FID artifacts, which allowed us to study the ***B***_1_ dependency of the CSF *T*_2_ mapping sequence. ***B***_0_ and ***B***_1_ maps were acquired, with use of identical resolution and FOV as for the *T*_2_ mapping acquisitions. The ***B***_0_ map was obtained with a gradient echo sequence with two different TEs (1.64 and 2.64 ms). The ***B***_1_ mapping sequence was based on the actual flip angle method [16], with first repetition time (TR) of 40 ms, second TR of 160 ms, TE of 0.96 ms, and a flip angle of 50°.

### In vivo measurements

**Fig. 2:**
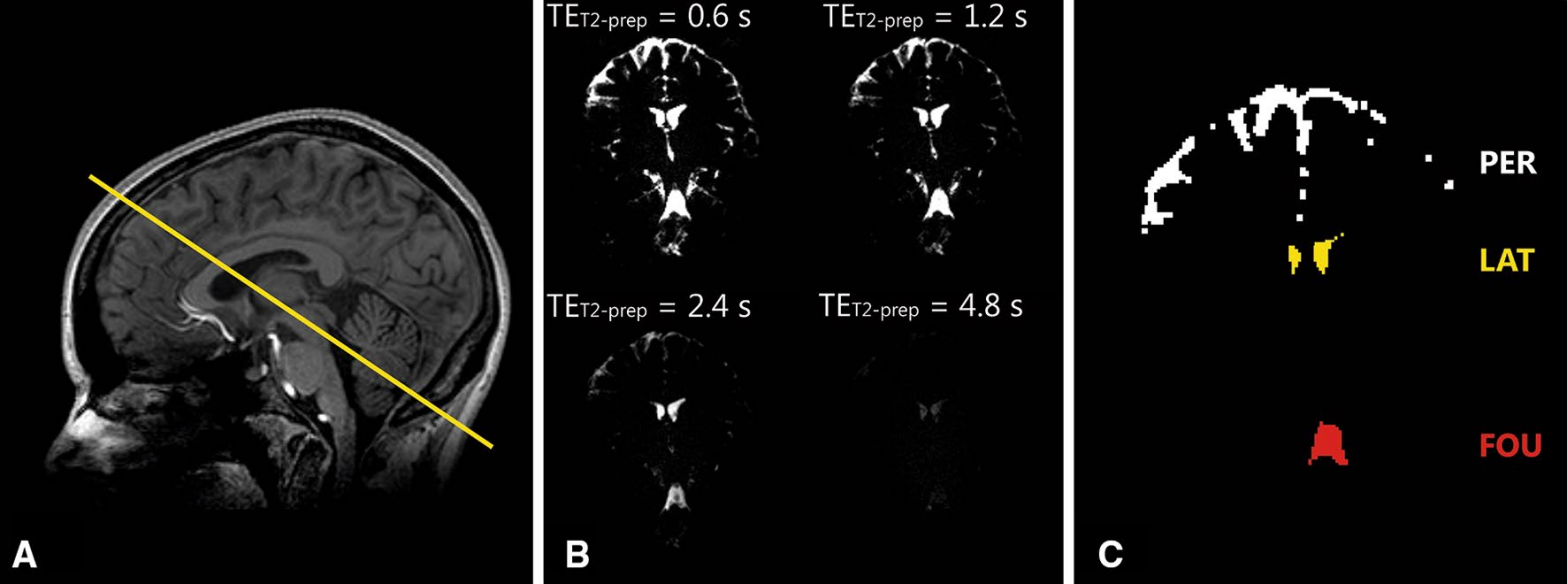
**a** Planning of the cerebrospinal fluid (CSF) *T*_2_ mapping scans, through the lateral ventricles and the fourth ventricle. **b** CSF *T*_2_ mapping scans at 7 T for increasing echo times (TE_T2-prep_) (0.6, 1.2, 2.4, and 4.8 s), shown with equal intensity scaling. **c** The region of interest masks used: the periphery (PER; white), the lateral ventricles (LAT; yellow), and the fourth ventricle (FOU; red)

In vivo experiments were performed at both 7 and 3 T to test the feasibility of CSF *T*_2_ mapping at 7 T, to further assess the sensitivity to ***B***_1_ inhomogeneities, and to explore the influence of partial volume effects. Seven healthy volunteers (three men, mean age 34 ± 11 years, age range 21–54 years) participated in this study. Informed consent was given by all volunteers in accordance with the requirements of the Institutional Review Board of the University Medical Center Utrecht (Utrecht, Netherlands). All volunteers were scanned with both a 3-T Philips Achieva scanner with an eight-channel head coil (Philips Healthcare, Best, Netherlands) and the 7-T scanner that was also used for the phantom study. The CSF *T*_2_ mapping scans were acquired in a single coronal slice, planned through both the lateral ventricles and the fourth ventricle (Fig. 2a). The scanning parameters are summarized in Table 1. The fixed *T*_delay_ was 15 s, and TR ranged between 20 and 25 s depending on TE_T2-prep_. Other parameters were as follows: SENSE factor 2.3 in the left–right direction and FOV of 240 × 240 mm^2^. Because of the long TR, the specific absorption rate (SAR) remained well within the specific absorption rate limits, also at 7 T. No additional methods were used to correct the scans for eddy currents. The low bandwidth of the scan may cause distortions in areas with poor shimming, such as the nasal cavities. However, shimming was good in the selected coronal slice. Also ***B***_0_ and ***B***_1_ maps were acquired.

**Table 1:** Scan parameters used for the in vivo experiments for the cerebrospinal fluid *T*_2_ mapping sequence (based on *T*_2_ preparation)

	Resolution (mm^3^)	TE_readout_ (ms)	TE_T2-prep_ (ms)	EPI factor	Bandwidth (phase/frequency) (Hz/voxel)	Scan duration (min)
3 T	1 × 1 × 4	133	0–4800^a^	105	8.1/961	2:59
	3 × 3 × 6	42	0–4800^a^	67	28.2/2308	2:59
7 T	1 × 1 × 2	127	0–4800^a^	107	8.4/1082	3:04
	1 × 1 × 4	126	0–4800^a^	107	8.4/1082	3:04
	3 × 3 × 6	23	0–4800^a^	37	55.1/2642	3:04

*EPI* echo planar imaging, *TE* echo time, *TE*_T2-prep_ echo time of T_2_-preparation

^a^The TE_T2-prep_ values used were 0, 600, 1200, 2400, and 4800 ms.

### Data analysis

#### Phantom

The resulting *T*_2_ estimates were analyzed as a function of ***B***_1_ and ***B***_0_. ***B***_1_ sensitivity was assessed with use of the scans with the highest ***B***_0_ gradient strength (0.5 mT/m), where no FID artifacts were present. The ***B***_1_ range present in the scan was used. On the basis of ***B***_1_ in each voxel, the voxels were sorted over eight bins of 5% ***B***_1_, leading to ***B***_1_ bins ranging from (85 ± 2.5)% to (120 ± 2.5)%. The signal was averaged over each ***B***_1_ bin, and *T*_2_ values were fitted over this averaged signal. ***B***_0_ sensitivity was assessed with the various applied ***B***_0_ gradient strengths (0, 0.05, 0.1, 0.2, 0.3, and 0.5 mT/m). In each scan, only voxels with ***B***_1_ between 97.5% and 102.5% were included. An additional intensity threshold was applied on the scan with the longest TE_T2-prep_ to minimize the influence of artifacts in the lower ***B***_0_ gradient scans. This threshold was set at 75% of the maximum intensity for the longest TE. The signal of all voxels included was averaged over each scan, and *T*_2_ values were fitted over this averaged signal.

#### In vivo

Three regions of interest (ROIs) were defined on the acquired in vivo scans: the lateral ventricles, the fourth ventricle, and peripheral CSF. The ROI masks were made by our applying an intensity threshold to the first TE (TE_T2-prep_ = 0.6 s). The intensity threshold was set at 25% of the maximum intensity in the image. Figure 2b and c shows the acquired CSF *T*_2_ mapping scan at 7 T with a resolution of 1 × 1 × 4 mm^3^ at all TEs for one volunteer, and the ROIs used. Conservative ROIs were used in the ventricles by our eroding the intensity-based ROIs with one voxel to minimize both partial volume and motion sensitivity. Erosion of the peripheral ROIs was not feasible.

The signal was averaged over each ROI, and *T*_2_ values were fitted over this averaged signal, with use of a single exponential decay model. Also mean ***B***_0_ and ***B***_1_ values were determined for each ROI. To minimize the influence of, for example, motion or partial volume effects on the data analysis, only fit results with *R* ^2^ of 0.99 or higher were considered.

#### Partial volume assessment

In the peripheral CSF an additional assessment of the influence of partial volume was made by our performing a partial volume correction. Only TE_T2-prep_ values of at least 1200 ms (excluding the shortest TE_T2-prep_ of 600 ms) were taken into account in the analysis. Thereby, maximal nulling of, for example, tissue signal was achieved, since the *T*_2_ values of tissue are below 100 ms [17, 18], about ten times shorter than the minimal TE_T2-prep_ used. The analysis with only the last TE_T2-prep_ times was also performed on the phantom scans to check for any systematic errors for all ***B***_0_ gradient strengths applied and ***B***_1_ between 97.5% and 102.5%.

All data analysis was performed in MATLAB (version 2015B, The MathWorks, Natick, MA, USA). IBM SPSS Statistics (version 21.0) was used for statistical analysis. Median *T*_2,CSF_ values and full ranges are reported. Wilcoxon signed-rank tests (significance level *p* < 0.05) were used to compare CSF T_2_ values in the lateral and fourth ventricles with those in the periphery to explore the observed *T*_2_ differences.

## Results

### Phantom measurements

**Fig. 3:**
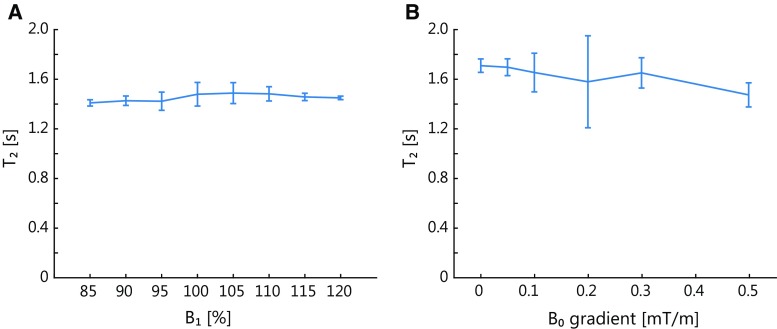
Results of the phantom measurements for the *B*_1_ (**a**) and ***B***_0_ gradient dependency (**b**) showing the fitted *T*_2_ for different ***B***_1_ values and through-plane ***B***_0_ gradient strengths, respectively. The error bars show the 95% confidence interval of the fitted *T*_2_. The cerebrospinal fluid *T*_2_ mapping sequence shows only minor sensitivity to ***B***_1_ and to the through-plane ***B***_0_ gradient (and thus to diffusion). The effect of the ***B***_0_ gradient was most apparent for the highest ***B***_0_ gradient. The ***B***_1_ dependency was determined with a ***B***_0_ gradient strength of 0.5 mT/m to avoid free induction decay (FID) artifacts in regions with ***B***_1_ deviating from 100%. For a ***B***_0_ gradient of 0.2 mT/m, the confidence interval was greater because of FID artifacts

Figure 3 shows the results of the phantom measurements for the ***B***_1_ dependency (Fig. 3a) and ***B***_0_ gradient dependency (Fig. 3b). The CSF *T*_2_ mapping sequence measured a *T*_2_ of 1.71 s (95% confidence interval 1.66–1.76 s) for ***B***_1_ of (100 ± 2.5)% and ***B***_0_ gradient strength of 0 mT/m. The sequence showed only minor ***B***_1_ sensitivity (assessed in the scans with ***B***_0_ gradient strength of 0.5 mT/m), with *T*_2_ ranging from 1.41 s (95% confidence interval 1.38–1.43 s) at ***B***_1_ of (85 ± 2.5)% to 1.49 s (95% confidence interval 1.40–1.57 s) at ***B***_1_ of (105 ± 2.5)%. Also minor ***B***_0_ gradient dependency was observed.

### In vivo measurements

Thirty-five CSF *T*_2_ mapping scans were acquired, for both field strengths and the different resolutions. Per scan, three fits were made, one per ROI, resulting in a total of 105 fits (42 at 3 T, 63 at 7 T). On the basis of the strict requirement on minimum *R* ^2^, 14 fits were excluded (four at 3 T, ten at 7 T), which corresponds to 13% of the total number of fits (10% at 3 T, 16% at 7 T); see Table 2 for a detailed overview.

**Table 2:** Number of scans acquired and *T*_2_ fits performed, and the number of excluded *T*_2_ fits per region of interest

Resolution (mm^3^)	Scans	Fits	Excluded fits		
			Lateral ventricles	Fourth ventricle	Periphery
3 T					
1 × 1 × 4	7	21	0	1	0
3 × 3 × 6	7	21	2	1	0
Total	14	42	2	2	0
7 T					
1 × 1 × 2	7	21	0	1	0
1 × 1 × 4	7	21	1	3	0
3 × 3 × 6	7	21	1	4	0
Total	21	63	2	8	0

**Fig. 4:**
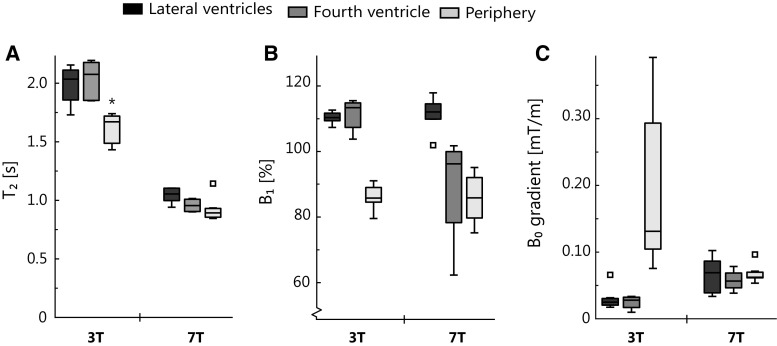
In vivo results: *T*_2_ (**a**), ***B***_1_ (**b**), and ***B***_0_ gradient (**c**) for the three different regions of interest. Outliers are represented by a square. Significant differences in measured *T*_2_ were found between the periphery and the lateral and fourth ventricles at 3 T (indicated by an asterisk)

The in vivo results for the scans with a resolution of 1 × 1 × 4 mm^3^ are summarized in Fig. 4. The results for the other resolutions were not significantly different from the data shown here (all data are shown in Tables S3, S4, S5). Although *T*_2_ differences between the resolutions were not significant, in most cases the shortest *T*_2_ times were observed for the largest voxel sizes.

At 7 T significantly shorter *T*_2_ times were found compared with at 3 T. At 3 T the *T*_2_ times measured in the periphery were significantly shorter than those measured in the lateral and fourth ventricles. The *T*_2_ times measured at 7 T were not significantly different between the three ROIs. At 3 T the median ***B***_1_ in the periphery was 85% (range 79–90%), while in the lateral and fourth ventricles it was 109% (ranges 106–112% and 103–114%). The median ***B***_0_ gradient in the periphery was 0.13 mT/m (range 0.07–0.38 mT/m), while in the lateral and fourth ventricles it was 0.02 and 0.03 mT/m, respectively (range 0.02–0.06 mT/m and 0.01–0.03 mT/m, respectively). At 7 T, lower ***B***_1_ values were observed in the periphery and the fourth ventricle [median 86% (range 75–94%) and 93% (range 62–101%), respectively], and higher ***B***_1_ values were observed in the lateral ventricles [median 111% (range 109–116)]. Similar ***B***_0_ gradients were observed in the three ROIs [median 0.07 mT/m (range 0.03–0.10 mT/m), 0.06 mT/m (range 0.04–0.08 mT/m), and 0.06 mT/m (range 0.05–0.09 mT/m), for the lateral ventricles, the fourth ventricle, and the periphery, respectively].

### Partial volume assessment

**Fig. 5:**
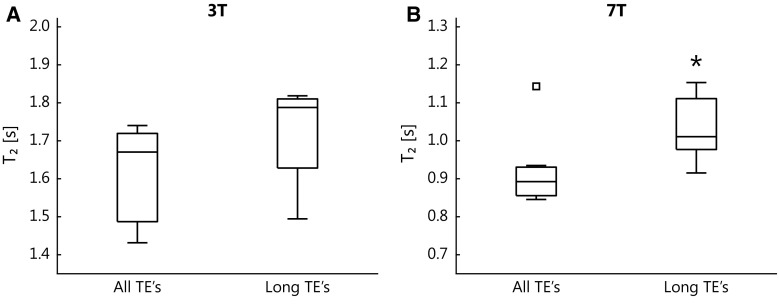
*T*_2_ values of peripheral cerebrospinal fluid resulting from the use of only the longest echo times (TEs) compared with the original analysis. Outliers are represented by a square. At both 3 and 7 T an increase in *T*_2_ can be observed. The asterisk indicates a significant difference with the original analysis (including all TEs)

**Fig. 6:**
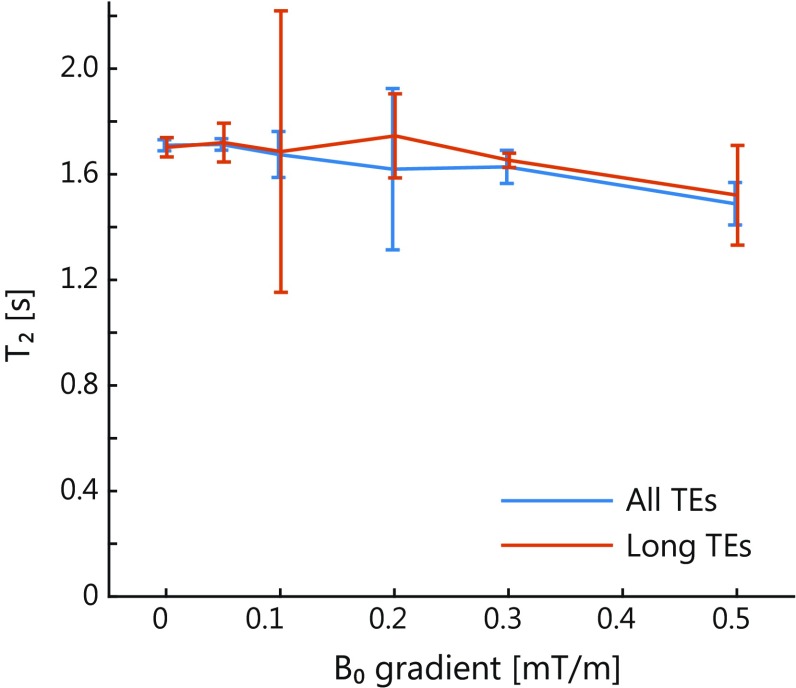
*T*_2_ values of the phantom, resulting from the use of only the longest echo times (TEs; orange) compared with the original analysis (blue). Both analyses result in similar *T*_2_ values

Figure 5 shows the results for the additional analysis of peripheral CSF to assess the influence of partial volume. The partial volume correction resulted in longer *T*_2_ times, with a significant increase of 118 ms at both 3 and 7 T. At 7 T the corrected peripheral CSF T_2_ was quite similar to the ventricular *T*_2_ (1.01 s vs 1.05 s), while at 3 T the mean peripheral T_2_ was still approximately 200 ms shorter than the ventricular *T*_2_ times [1.79 s (range 1.49–1.82 s) vs 2.03 s (range 1.73–2.16 s), *p* = 0.02]. The results for this analysis of the phantom data are shown in Fig. 6. Both analysis methods (including all TEs or only the longest TEs) resulted in similar *T*_2_ values, indicating no systematic errors in the additional analysis with only the longest TEs.

## Discussion

In this research we have shown the feasibility of CSF *T*_2_ mapping at 7 T with a dedicated CSF *T*_2_ mapping sequence based on *T*_2_ preparation, which was initially developed at 3 T. We investigated the sensitivity of this sequence for the influence of ***B***_1_, diffusion (***B***_0_ gradient), and partial volume effects. The sequence appeared to be relatively insensitive to ***B***_1_ and ***B***_0_ inhomogeneity. Partial volume effects tend to lower the observed *T*_2_ values at the periphery. *T*_2,CSF_ was considerably shorter at 7 T than at 3 T in all three ROIs. The peripheral *T*_2,CSF_ was significantly shorter than the ventricular *T*_2,CSF_ at 3 T (but not at 7 T).

The peripheral *T*_2,CSF_ increased considerably on partial volume correction, as obtained from analysis of long TEs (more than ten times the tissue *T*_2_). The partial volume correction for the SE-EPI sequence, which was used as a relatively ***B***_1_-insensitive reference (data shown in the electronic supplementary material), did not significantly increase *T*_2_ values, although the SE-EPI sequence showed an even larger *T*_2_ difference between the periphery and ventricles. The ventricular *T*_2_ values measured with the CSF *T*_2_ mapping sequence at 3 T match with *T*_2_ values found in literature [1, 10, 19, 20]. Given the results from our measurements and analysis, we believe that the observed *T*_2_ difference between the ventricular and peripheral CSF could be partly due to physiological differences. However, the different results for different sequences and field strengths and the confounding influence of partial volume effects will make it challenging to accurately isolate and quantify any true physiological effect from confounders. This will hamper applications in research focusing on in vivo evaluation of the (regional) composition of CSF.

### ***B***_1_ and ***B***_0_ dependency

In the phantom measurements only minor ***B***_1_ dependency was found for the CSF *T*_2_ mapping sequence, as shown in Fig. 3a. Also, the measurements with an increasing through-plane ***B***_0_ gradient showed only limited ***B***_0_ gradient dependency, except for the highest ***B***_0_ gradient (0.5 mT/m), as shown in Fig. 3b. In the in vivo measurements, the ***B***_0_ gradient was similar between the periphery and the ventricles at 7 T, and differed by a maximum of 0.38 mT/m (median ***B***_0_ gradient was 0.13 mT/m) at 3 T (Fig. 4c). This difference in ***B***_0_ homogeneity between 3 and 7 T is probably due to different shimming techniques: image-based third-order shimming was used at 7 T, and linear shimming was used at 3 T. The low sensitivity to ***B***_0_ gradient shows that the *T*_2_ mapping sequence is relatively insensitive to diffusion. It is not likely that ***B***_0_ gradients due to imperfect shimming contributed considerably to the observed difference in *T*_2_ between periphery and ventricles.

### Partial volume effects

The different resolutions used at both field strengths did not yield considerably different *T*_2_ values (Tables S3, S4, S5), although there is a trend of longer measured ventricular *T*_2_ times for smaller voxel sizes at 3 T, similarly to what was found by to De Vis et al. [10]. As the ventricular ROIs were eroded, the voxels at the edges, where more partial volume is expected, were discarded. For the periphery, however, erosion was not feasible because of the thin shape of the ROI. Moreover, the ROI definition was based on an intensity threshold, which depends on the CSF fraction in each voxel. Since the total subarachnoidal CSF volume is quite small, and distributed over a relatively large area [21], partial volume is probably present in all peripheral ROIs, independently of the voxel sizes used in this work.

The role of partial volume effects regarding the measured peripheral *T*_2,CSF_ was investigated by use of the longest TEs only (Fig. 5) to maximally remove the influence of partial volume. It could seem unexpected that the use of the late TEs reveals a considerable partial volume effect, since the first TE_T2-prep_ is already relatively long compared with the tissue *T*_2_. The *T*_2_ of gray matter is approximately 90 ms at 3 T [18, 22] and 55 ms at 7 T [18, 23], while the first TE was 600 ms. However, it is possible that partial volume occurs with a compartment with a relatively long *T*_2_ in the cerebral cortex, like arterial blood (*T*_2_ around 150 ms at 3 T [24, 25]) or the outer rim of the cortex (unknown but long *T*_2_, greater than 100 ms, at 7 T [26]). At the shortest TE_T2prep_ (600 ms), the signal of arterial blood has decayed to 2%. However, in the case of small partial volume fractions of CSF in the periphery, this could still have a considerable influence on the measured *T*_2_. The outer layer of the cerebral cortex (layer I) may have a long *T*_2_ because it contains almost no neuronal cell bodies, and many glial cells instead, similarly to gliotic lesions, which also have a long *T*_2_ [26].

### Peripheral versus ventricular CSF *T*_2_ and field strength dependence

De Vis et al. [10] found a *T*_2_ difference of 609 ± 133 ms between the periphery and the ventricles at 3 T, and Qin found a *T*_2_ difference of 420 ± 155 ms at 3 T. Also in this work a shorter *T*_2,CSF_ was measured in the periphery compared with the ventricles, as shown in Fig. 4a. This *T*_2_ difference is larger at 3 T than at 7 T: the *T*_2_ difference is 365 ms at 3 T and 161 ms at 7 T, which corresponds to differences of 18% and 15% relative to the *T*_2_ in the lateral ventricles for 3 and 7 T, respectively. Partial volume correction, which led to a peripheral CSF *T*_2_ increase of 118 ms at both field strengths (Fig. 5), resulted in remaining *T*_2_ differences of 247 ms and only 43 ms for 3 and 7 T, respectively. These correspond to a *T*_2_ difference of 12% and 4% relative to the ventricular CSF *T*_2_ for 3 and 7 T, respectively. A relatively larger *T*_2_ difference was found when a SE-EPI sequence was used, and remained largely unchanged after partial volume correction (data shown in the electronic supplementary material).

These results indicate a true *T*_2_ difference between peripheral and ventricular CSF. A potential physiological explanation for this observed *T*_2_ difference could be sought in differences in, for example, in the levels of O_2_, protein, and/or glucose, since these substrates are known to decrease *T*_2_ [20, 27–29]. However, relatively large concentration differences are necessary to bridge the difference between peripheral and ventricular *T*_2,CSF_. So although differences in CSF composition may partly cause the observed *T*_2_ difference, it seems unlikely that these are the only contributor.

The shorter in vivo CSF *T*_2_ at 7 T than at 3 T (Fig. 4a) is in line with published in vivo measurements by Daoust et al. [20]. However, Daoust et al. suggested that the *T*_2_ of CSF is not field strength dependent, but that residual field gradients cause errors in in vivo measurements at higher field strengths. If the *T*_2_ measurements are strongly dependent on residual gradients, one might expect that the *T*_2_ difference between periphery and ventricles observed at 3 T is also largely due to residual field gradients, such as ***B***_0_ gradients. However, the CSF *T*_2_ mapping sequence used in our study showed negligible ***B***_0_ gradient dependency for the measured *T*_2_ up to 0.3 mT/m, while the observed ***B***_0_ gradients in the brain were between 0.07 and 0.38 mT/m, and on average well below 0.20 mT/m. The limited diffusion sensitivity of the CSF *T*_2_ mapping sequence is also visible from the results of the long TE analysis on the phantom measurements. The measured *T*_2_ remained unchanged when only long TEs (with stronger diffusion weighting) were used (see Fig. 6).

### Implications

Before CSF *T*_2_ mapping can be used as a parameter to study diseases such as cerebral small vessel disease, several uncertainties need to be resolved. It is not yet clear to what extent the *T*_2_ difference between ventricular and peripheral CSF reflects physiological differences in CSF composition. The CSF *T*_2_ mapping sequence shows a much smaller *T*_2_ difference compared with SE-EPI, while the difference also varies with field strength. Overall, the *T*_2_ difference between peripheral and ventricular CSF could (partly) be explained by (a combination of) physiological differences. The possibility that the shorter peripheral *T*_2_ is entirely caused by an artifact, like *B*_0_ gradients caused by imperfect shimming and/or partial volume effects between tissue, blood, and CSF, seems unlikely.

Care should be taken when one is interpreting *T*_2_ measurements of CSF, and more work is necessary to find the true explanations for the *T*_2_ differences between 3 and 7 T and between the peripheral and ventricular CSF at 3 T.

### Limitations

The major limitation of this work is that it is an observational study, which limits the extent to which underlying mechanisms causing the observations can be identified. Despite our efforts to separate the effects of partial volume and true physiological differences, it remains uncertain to what extent the observed shorter peripheral *T*_2,CSF_ is due to different CSF compositions.

Furthermore, the statistical power of this study was limited by the low number of volunteers combined with the stringent *R* ^2^ criterion, which resulted in a relatively large dropout of ROIs.

Moreover, only macroscopic ***B***_0_ gradients could be determined in the in vivo scans, and the magnitude of microscopic, subvoxel ***B***_0_ gradients remains unknown.

Finally, no in vitro CSF sample was used to validate the in vivo measurements. In vitro CSF is prone to changes in, for example, O_2_ content, compared with in vivo CSF, which may induce *T*_2_ differences between in vitro and in vivo CSF.

## Conclusion

CSF *T*_2_ mapping with a dedicated sequence is feasible at both 3 and 7 T, and yields shorter CSF *T*_2_ times at 7 T compared with 3 T. At 3 T, shorter *T*_2_ times were found for peripheral CSF compared with ventricular CSF; at 7 T this effect was much smaller. Partial volume effects can partly explain this *T*_2_ difference, but a physiological contribution to the difference in *T*_2_ between ventricular and peripheral CSF is possible. The different results for different sequences and field strengths, and the confounding influence of partial volume, will make it challenging to accurately isolate and quantify any true physiological effect for applications in research focusing on in vivo evaluation of the (regional) composition of CSF.

## Electronic supplementary material

Below is the link to the electronic supplementary material.

Supplementary material 1 (PDF 321 kb)
