# Supplementary material for: T2 mapping of cerebrospinal fluid: 3 T versus 7 T
Source: MAGMA. 2017 Nov 6;31(3):415–24. doi: 10.1007/s10334-017-0659-3 (PMC5973950; doi:10.1007/s10334-017-0659-3)
Supplement: Supplementary file 1 — Supplementary material 1 (PDF 321 kb) [file 10334_2017_659_MOESM1_ESM.pdf]

***$T_2$ mapping of cerebrospinal fluid: 3T versus 7T***

*Magnetic Resonance Materials in Physics, Biology and Medicine*

Jolanda M Spijkerman, Esben T Petersen, Jeroen Hendrikse, Peter Luijten, Jaco JM Zwanenburg

*Corresponding author:*

J.M. Spijkerman, MSc

Department of Radiology

University Medical Center Utrecht

Utrecht, The Netherlands

Telephone: +31-(0)88-75560297

E-mail: j.m.spijkerman-2@umcutrecht.nl

***$T_2$ mapping with a single echo SE-EPI sequence with various TEs as truly $B_1$ insensitive reference for the CSF $T_2$ mapping sequence***

## Methods

### SE-EPI sequence

As a reference for the CSF $T_2$ mapping method a single echo SE-EPI sequence was used, equal to the readout used in the CSF $T_2$ mapping sequence, but with increasing (very) long TEs (Figure S1). Crushers were applied before and after the refocusing pulse to crush the free induction decay signal from the refocusing pulse in case of an imperfect 180° pulse (inhomogeneous $B_1$). To minimize motion sensitivity, some modifications were made: the slice rewinder gradient was applied directly after the slice excitation pulse, while the phase encoding gradient was applied just prior to the EPI readout train. The motion sensitivity of the crusher gradients around the 180° refocusing pulse is limited, due the relatively slow flow of CSF (around 2-4 mm/s [1]) and the short spacing between both crusher lobes. The shortest used TE was aimed to be shorter than the first non-zero TE for the CSF $T_2$ mapping sequence, since the SE-EPI sequence has a higher diffusion sensitivity. The longest TE was aimed to be in the same range as the longest TE for the CSF $T_2$ mapping sequence. Therefore the TEs were heuristically defined according to the following formula: $TE = 240 + 45 \cdot n^2$, for the $n^{th}$ acquisition. This resulted in the following TEs : 240, 285, 420, 645, 960, 1365, 1860, 3120, 3885, and 4740 ms. After the readout a fixed delay time ($T_{delay}$) was applied. The other parameters are specified at the description of the in vivo measurements.

### Phantom experiment

A single slice was acquired with 3x3x6 $mm^3$ resolution, FOV 240x96 $mm^2$, sensitivity encoding (SENSE) [2] factor 1, and TR of 15 s, resulting in a $T_{delay}$ of 14.7 s. A series of $T_2$ maps with increasing through-plane $B_0$ gradients was acquired to study the effect of diffusion for both sequences for $B_1$ 100%. The following through-plane $B_0$ gradient strengths were applied by adding this strength to the linear shim term in the user interface: 0, 0.05, 0.1, 0.2, 0.3, and 0.5 mT/m. The acquisition with the highest $B_0$

gradient was used to study the $B_1$ dependency of the SE-EPI sequence, similar to the CSF $T_2$ mapping sequence.

### In vivo experiment

The SE-EPI scan was acquired in all seven volunteers, in a single coronal slice (identical slice as the CSF $T_2$ mapping sequence). The scanning parameters are summarized in Supplementary table S1. Shared parameters were: SENSE factor 2.3 in left-right direction, FOV 240×240 $mm^2$. The fixed $T_{delay}$ after image acquisition was 14.7 s and TR varied between 15-19s, depending on TE.

### Data analysis

Data analysis was identical to the data analysis of the CSF $T_2$ mapping sequence.

The in vivo ROI masks were made by applying an intensity threshold (25% of the maximum intensity in the scan) to the first echo time for the SE-EPI sequence ($TE_{SE\text{-}EPI}$ = 0.24 s), leading to similar ROIs compared with the CSF $T_2$ mapping sequence.

For the partial volume assessment only TEs of at least 960 ms were taken into account in the analysis. The minimum TE of 960 ms is shorter than the minimum TE of 1200 ms for the CSF $T_2$ mapping sequence, since shorter $T_2$ values were expected to be found for the SE-EPI sequence due to higher diffusion sensitivity.

## Results

### Phantom measurements

Supplementary figure S2 shows the results of the phantom measurements for the $B_1$ (Figure S2A) and $B_0$ gradient (Figure S2B) dependency. For $B_1$ = 100 ± 2.5% and $B_0$ gradient = 0 mT/m, the $T_2$ value obtained with the SE-EPI sequence was 0.96 s (95% CI: [0.86-1.05]). The SE-EPI sequence showed no $B_1$ dependency, but considerably shorter $T_2$ values were found for increasing $B_0$ gradients.

### In vivo measurements

In one volunteer the SE-EPI scan at 3T (both resolutions) could not be acquired due to time constraints. A total of 33 SE-EPI scans was acquired, for both field strengths, and the different resolutions. Per scan 3 fits were made, one per ROI, resulting in a total of 99 fits (36 at 3T, 63 at 7T). Based on the strict requirement on minimum $R^2$, 21 fits were excluded (8 at 3T, 13 at 7T), which is 21% of the total number of fits (22% at 3T, 21% at 7T), see Supplementary table S2 for a detailed overview.

The in vivo results for the SE-EPI scans with resolution 1×1×4 $mm^3$ are summarized in supplementary Figure S3. At both 3T and 7T the $T_2$s measured in the periphery were significantly shorter compared to only the lateral ventricles. In all ROIs and for both field strengths, considerably shorter $T_2$s were measured with the SE-EPI sequence compared to the CSF $T_2$ mapping sequence. The results for the other resolutions were not significantly different from the data shown here (all data, for both the SE-EPI sequence and the CSF $T_2$ mapping sequence, is shown in supporting tables S3, S4, and S5).

### Partial volume assessment

The results for the additional analysis of peripheral CSF with the longest TEs only are shown in supplementary Figure S4. At 3T, the peripheral CSF $T_2$ increased with 83 ms at 3T, although this was not significant. At 7T, no difference was observed between the corrected and uncorrected peripheral CSF $T_2$.

The results for this analysis for the phantom data resulted in shorter $T_2$ values (Supplementary figure S5). This $T_2$ shortening was larger for larger $B_0$ gradients.

## Discussion

For the SE-EPI sequence the peripheral $T_{2,CSF}$ was significantly shorter than the ventricular $T_{2,SCF}$ at both 3T and 7T.

### $B_1$ and $B_0$ dependency

In the phantom measurements no $B_1$ dependency was found for the SE-EPI sequence, similar to the CSF $T_2$ mapping sequence, as shown in Supplementary figure S2. The measurements with an increasing through-plane $B_0$ gradient resulted in shorter $T_2$ values for larger $B_0$ gradients. Only a relatively small gradient was needed to find shorter $T_2$ values for the SE-EPI sequence, while a much larger gradient was needed to find shorter $T_2$ values for the CSF $T_2$ mapping sequence (shown in Figure 3B). This can be explained by the longer echo spacings compared to the CSF $T_2$ mapping sequence, which increases the sensitivity for diffusion [3]. Higher diffusion sensitivity for the SE-EPI sequence is also apparent from the analysis including only the longest TEs: shorter $T_2$ values were obtained for the SE-EPI sequence when only the longest echo spacings (longest TEs) were included. In contrast, the CSF $T_2$ mapping sequence showed similar results when only the longest TEs were included, for all $B_0$ gradient strengths. The difference in diffusion sensitivity of both sequences, is further illustrated by the relative difference in b-value for both sequences which can be readily computed from the echo spacing and the number of refocusing pulses [4]: the b-value of the SE-EPI sequence is 20 to 986 times larger compared with the CSF $T_2$ mapping sequence, for TEs 600-4800 ms, respectively. The difference in diffusion sensitivity is also visible in the different behavior of the long TE analysis on the phantom measurements. For the SE-EPI a shorter $T_2$ is measured if only long TEs (with stronger diffusion weighting) is used, but this is not the case for the CSF $T_2$ mapping sequence (compare Figure 6 with supplementary Figure S5). The difference in diffusion sensitivity is also visible in the different behavior of the long TE analysis on the phantom measurements. For the SE-EPI a shorter $T_2$ is measured if only long TEs (with stronger diffusion weighting) is used, but this is not the case for the CSF $T_2$ mapping sequence (compare Figure 6 with supplementary Figure S5).

**Partial volume effects**

Partial volume correction showed a relatively small $T_2$ increase (approximately 80 ms) at 3T, but this was not significant. At 7T the $T_2$ values did not change. This suggests that for the SE-EPI sequence the observed $T_2$ difference between ventricular and peripheral CSF is (predominantly) not caused by partial volume effects. However, the $T_2$ values measured with this sequence are much shorter compared to

the CSF $T_2$ mapping sequence, probably due to its high sensitivity for diffusion and flow. If the partial volume effect in the CSF $T_2$ mapping sequence is indeed caused by arterial blood or relatively free water in the outer rim of the cortex, the diffusion sensitivity of the SE-EPI may decrease the sensitivity for partial volume effects from this compartment. Alternatively, it could be that the microscopic gradients around the (venous) vasculature at the brain surface induce a shorter $T_2$ due to the diffusion sensitivity of the SE-EPI sequence, and that this effect cannot be corrected for by choosing longer TEs.

**Peripheral versus ventricular CSF $T_2$ and field strength dependence**

The $T_2$ difference between peripheral and ventricular CSF was 517 ms at 3T, and 160 ms at 7T, a relative difference of 40% and 26% compared to the $T_2$ in the lateral ventricles. After partial volume correction, the $T_2$ difference decreased to 433 ms for 3T, a difference of 33% relative to the ventricular CSF $T_2$, and was unchanged at 7T. Thus, a much larger $T_2$ difference between peripheral and ventricular CSF was found compared with the CSF $T_2$ mapping sequence. The $T_2$ difference at 3T can be partly explained by the larger $B_0$ gradient in the periphery, but at 7T the $B_0$ gradients are similar $B_0$ gradients were found for all ROIs. Furthermore, overall shorter $T_2$ values were found compared with the CSF $T_2$ mapping sequence. The SE-EPI sequence is more sensitive to flow and diffusion than the CSF $T_2$ mapping sequence, which uses multiple refocusing pulses with a fixed echo spacing that is shorter than the shortest TE used in the SE-EPI sequence. The higher flow and diffusion sensitivity of SE-EPI might (partly) cause the larger $T_2$ difference between peripheral and ventricular CSF. The CSF flow in the periphery is lower compared to the ventricles and cannot explain a shorter peripheral $T_2$, but diffusion effects around the blood vessels on the cortex may be relatively strong. Since the ventricular walls are not covered with blood vessels, diffusion effects may be smaller in the ventricles in areas away from the choroid plexus at the base of the ventricles. Based on Kiselev, et al. [5], the level of signal dephasing due to diffusion around blood vessels is larger at 7T compared to 3T. This would imply a larger difference between peripheral and ventricular $T_2$ at 7T, contrary to our observations. It is conceivable, however, that the relative contribution of macroscopic field inhomogeneity to diffusion related $T_2$ shortening is larger at 7T than at 3T for the SE-EPI $T_2$ mapping, which could partially mask

regional differences in microscopic field inhomogeneity. This is, however, not supported by the measured $B_0$ gradients from the $B_0$ maps that were acquired in vivo. As we used image based third order shimming at 7T, the shimming at the periphery seemed to be better than the shimming at 3T, which was linear shimming.

**Limitations**

The SE-EPI sequence resulted in different $T_2$s compared with the CSF $T_2$ mapping sequence, partly due to a different sensitivity to e.g. flow and diffusion effects. The effects of these confounding factors on the observed $T_2$ were not studied thoroughly in this work.

Moreover, for the phantom measurements with increasing $B_0$ background gradient, the acquired signal decay profile showed some deviation from a single exponential decay profile due to relatively stronger diffusion effects for longer TEs. For the in vivo data however, the acquired signal decay only showed minor deviation from a single exponential decay profile. Some in vivo scans were excluded based on the minimum $R^2$ of 0.99, this was mainly due to motion and/or the presence of flow voids in the data.

Finally, for the SE-EPI sequence at 7T, the measured peripheral CSF $T_2$ was 450 ms. For the partial volume correction a minimum TE of 960 ms was used, almost double the CSF $T_2$. This may decrease the sensitivity for small $T_2$ changes.

## References


1. Sweetman B, Linninger AA (2011) Cerebrospinal fluid flow dynamics in the central nervous system. Ann Biomed Eng 39:484–496.

2. Pruessmann KP, Weiger M, Scheidegger MB, Boesiger P (1999) SENSE: Sensitivity encoding for fast MRI. Magn Reson Med 42:952–962.

3. Dale BM, Brown MA, Semelka RC (2015) MRI Basic principles and applications, Fifth edit. John Wiley & Sons Inc

4. Carr H, Purcell E (1954) Effects of Diffusion on Free Precession in Nuclear Magnetic Resonance Experiments. Phys Rev 94:630–638.

5. Kiselev V, Posse S (1998) Analytical model of susceptibility induced MR signal dephasing in a microvascular network. Proc. Intl. Soc. Mag. Reson. Med. p 1397

# Supplementary tables

## Supplementary table S1: Scan parameters

Scan parameters used for the in vivo experiments for the SE-EPI sequence

| | Resolution | $TE_{readout}$ [ms] | EPI factor | BW (phase/freq) [Hz/voxel] | Scan dur. [min] |
|---|---|---|---|---|---|
| **3T** | 1×1×4 | 240-4740 [1] | 105 | 8.3 / 978 | 3:30 |
| | 3×3×6 | 240-4740 [1] | 67 | 23.7 / 1806 | 3:30 |
| **7T** | 1×1×2 | 240-4740 [1] | 107 | 8.5 / 1103 | 3:30 |
| | 1×1×4 | 240-4740 [1] | 107 | 8.5 / 1103 | 3:30 |
| | 3×3×6 | 240-4740 [1] | 37 | 56.3 / 2626 | 3:30 |

[1] The used TEs were: 240, 285, 420, 645, 960, 1365, 1860, 2445, 3120, 3885, 4740 ms

**Supplementary table S2: Number of excluded fits**

Number of acquired scans and performed $T_2$ fits and the number of excluded $T_2$ fits per ROI, for the SE-EPI sequence

| | | Scans (fits) | Excluded fits | | |
|---|---|---|---|---|---|
| | | | *Lateral ventricles* | *Fourth ventricle* | *Periphery* |
| **3T** | 1×1×4 | 6 (18) | 1 | 1 | 0 |
| | 3×3×6 | 6 (18) | 1 | 5 | 0 |
| *Total* | | *12 (36)* | *2* | *6* | *0* |
| **7T** | 1×1×2 | 7 (21) | 0 | 2 | 0 |
| | 1×1×4 | 7 (21) | 1 | 1 | 1 |
| | 3×3×6 | 7 (21) | 3 | 4 | 1 |
| *Total* | | *21 (63)* | *4* | *7* | *2* |

**Supplementary table S3: In vivo results, lateral ventricles**

$T_2$, $B_1$, and $B_0$ values for the lateral ventricles, and the ROI sizes and number of included fits, n, for both the CSF $T_2$ mapping sequence ($T_2$-prep) and the SE-EPI sequence

| | | | **$T_2$ [s] median (mean±std)** | **$B_0$ gradient [mT/m] median (mean±std)** | **$B_1$ [%] median (mean±std)** | **ROI size [$mm^2$] median (mean±std)** | **n** |
|---|---|---|---|---|---|---|---|
| 3T | $T_2$-prep | 1×1×4 | 2.03 (1.99 ± 0.16) | 0.025 (0.029 ± 0.016) | 109 (109 ± 2) | 122 (128 ± 85) | 7 |
| | | 3×3×6 | 1.96 (2.02 ± 0.10) | 0.032 (0.034 ± 0.017) | 111 (111 ± 1) | 54 (68 ± 54) | 5 |
| | SE-EPI | 1×1×4 | 1.28 (1.30 ± 0.11) | 0.024 (0.023 ± 0.004) | 108 (108 ± 2) | 127 (148 ± 101) | 5 |
| | | 3×3×6 | 1.13 (1.16 ± 0.24) | 0.029 (0.036 ± 0.023) | 109 (109 ± 2) | 54 (65 ± 57) | 5 |
| 7T | $T_2$-prep | 1×1×2 | 1.07 (1.12 ± 0.17) | 0.059 (0.063 ± 0.028) | 110 (108 ± 8) | 98 (103 ± 95) | 7 |
| | | 1×1×4 | 1.05 (1.04 ± 0.07) | 0.068 (0.066 ± 0.027) | 111 (110 ± 5) | 87 (83 ± 54) | 6 |
| | | 3×3×6 | 1.06 (1.04 ± 0.07) | 0.062 (0.069 ± 0.034) | 113 (110 ± 9) | 59 (63 ± 55) | 6 |
| | SE-EPI | 1×1×2 | 0.64 (0.66 ± 0.13) | 0.057 (0.066 ± 0.038) | 110 (110 ± 7) | 123 (115 ± 96) | 7 |
| | | 1×1×4 | 0.61 (0.65 ± 0.12) | 0.055 (0.064 ± 0.034) | 111 (109 ± 8) | 90 (111 ± 110) | 6 |
| | | 3×3×6 | 0.55 (0.59 ± 0.15) | 0.068 (0.076 ± 0.049) | 110 (109 ± 11) | 54 (65 ± 50) | 4 |

**Supplementary table S4: In vivo results, fourth ventricle**

$T_2$, $B_1$, and $B_0$ values for the fourth ventricle, and the ROI sizes and number of included fits, n, for both the CSF $T_2$ mapping sequence ($T_2$-prep) and the SE-EPI sequence

| | | | **$T_2$ [s] median (mean±std)** | **$B_0$ gradient [mT/m] median (mean±std)** | **$B_1$ [%] median (mean±std)** | **ROI size [$mm^2$] median (mean±std)** | **n** |
|---|---|---|---|---|---|---|---|
| 3T | $T_2$-prep | 1×1×4 | 2.08 (2.04 ± 0.16) | 0.028 (0.024 ± 0.009) | 112 (110 ± 5) | 75 (86 ± 29) | 6 |
| | | 3×3×6 | 1.99 (1.97 ± 0.19) | 0.029 (0.023 ± 0.012) | 112 (110 ± 5) | 14 (20 ± 13) | 6 |
| | SE-EPI | 1×1×4 | 1.30 (1.25 ± 0.32) | 0.027 (0.024 ± 0.011) | 112 (110 ± 5) | 79 (84 ± 26) | 5 |
| | | 3×3×6 | 1.47 (1.47 ± 0) | 0.004 (0.004 ± 0) | 100 (100 ± 0) | 27 (27 ± 0) | 1 |
| 7T | $T_2$-prep | 1×1×2 | 1.12 (1.14 ± 0.21) | 0.049 (0.049 ± 0.021) | 91 (86 ± 13) | 58 (58 ± 24) | 6 |
| | | 1×1×4 | 0.96 (0.96 ± 0.06) | 0.056 (0.057 ± 0.016) | 96 (89 ± 18) | 60 (64 ± 30) | 4 |
| | | 3×3×6 | 1.04 (1.14 ± 0.28) | 0.051 (0.047 ± 0.014) | 92 (93 ± 2) | 18 (21 ± 14) | 3 |
| | SE-EPI | 1×1×2 | 0.75 (0.74 ± 0.09) | 0.042 (0.044 ± 0.016) | 94 (92 ± 9) | 52 (56 ± 31) | 5 |
| | | 1×1×4 | 0.61 (0.62 ± 0.15) | 0.058 (0.052 ± 0.020) | 94 (88 ± 14) | 43 (52 ± 32) | 6 |
| | | 3×3×6 | 0.73 (0.73 ± 0.08) | 0.058 (0.047 ± 0.026) | 92 (91 ± 9) | 18 (18 ± 0 ) | 3 |

**Supplementary table S5: In vivo results, periphery**

$T_2$, $B_1$, and $B_0$ values for the periphery, and the ROI sizes and number of included fits, n, for both the CSF $T_2$ mapping sequence ($T_2$-prep) and the SE-EPI sequence. a) significant vs lateral ventricles, b) significant vs fourth ventricle

| | | | **$T_2$ [s] median (mean±std)** | **$B_0$ gradient [mT/m] median (mean±std)** | **$B_1$ [%] median (mean±std)** | **ROI size [$mm^2$] median (mean±std)** | **n** |
|---|---|---|---|---|---|---|---|
| 3T | $T_2$-prep | 1×1×4 | 1.67 (1.61 ± 0.13) [a,b] | 0.129 (0.194 ± 0.118) | 85 (86 ± 4) | 632 (664 ± 244) | 7 |
| | | 3×3×6 | 1.59 (1.60 ± 0.12) [a,b] | 0.094 (0.150 ± 0.143) | 87 (87 ± 4) | 1161 (1103 ± 329) | 7 |
| | SE-EPI | 1×1×4 | 0.76 (0.74 ± 0.10) [a] | 0.143 (0.187 ± 0.111) | 85 (85 ± 3) | 818 (774 ± 245) | 6 |
| | | 3×3×6 | 0.74 (0.72 ± 0.10) [a] | 0.114 (0.173 ± 0.123) | 86 (86 ± 3) | 1283 (1248 ± 352) | 6 |
| 7T | $T_2$-prep | 1×1×2 | 0.93 (0.94 ± 0.10) [b] | 0.062 (0.067 ± 0.014) | 85 (84 ± 7) | 421 (456 ± 119) | 7 |
| | | 1×1×4 | 0.89 (0.92 ± 0.10) | 0.061 (0.066 ± 0.014) | 85 (85 ± 7) | 484 (493 ± 109) | 7 |
| | | 3×3×6 | 0.91 (0.93 ± 0.10) | 0.074 (0.076 ± 0.015) | 85 (85 ± 6) | 936 (928 ± 224) | 7 |
| | SE-EPI | 1×1×2 | 0.46 (0.46 ± 0.08) [a,b] | 0.077 (0.080 ± 0.031) | 86 (86 ± 8) | 501 (493 ± 119) | 7 |
| | | 1×1×4 | 0.45 (0.45 ± 0.08) [a] | 0.080 (0.081 ± 0.028) | 88 (88 ± 7) | 462 (471 ± 122) | 6 |
| | | 3×3×6 | 0.43 (0.45 ± 0.08) | 0.077 (0.075 ± 0.015) | 86 (86 ± 5) | 684 (720 ± 207) | 6 |

## Supplementary figures

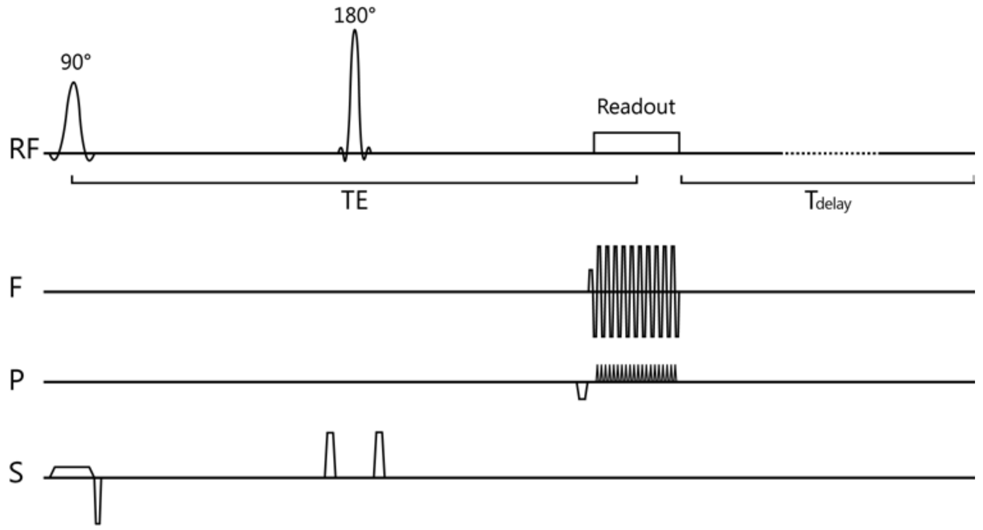


**Figure S1** Single echo SE-EPI pulse sequence. For $T_2$ mapping, the echo time TE was varied. The applied RF pulses are shown on the RF axis, the applied gradients are shown on the frequency (F), phase (P), and slice (S) encoding axes

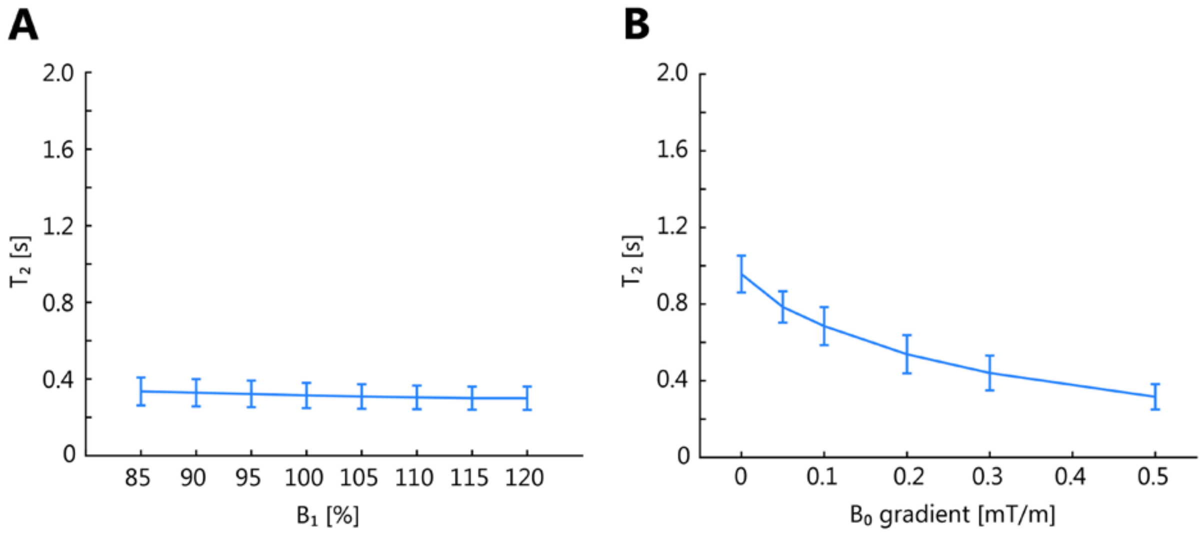


**Figure S2** Results of the phantom measurements for the $B_1$ (A) and $B_0$ gradient dependency (C), showing the fitted $T_2$s for different $B_1$s and through-plane $B_0$ gradient strengths, respectively. The error bars show the 95% confidence interval of the fitted $T_2$s. The SE-EPI sequence shows overall shorter $T_2$s than the CSF $T_2$ mapping sequence. The SE-EPI sequence is insensitive to $B_1$, but shows shorter $T_2$ values for increasing $B_0$ gradient strengths

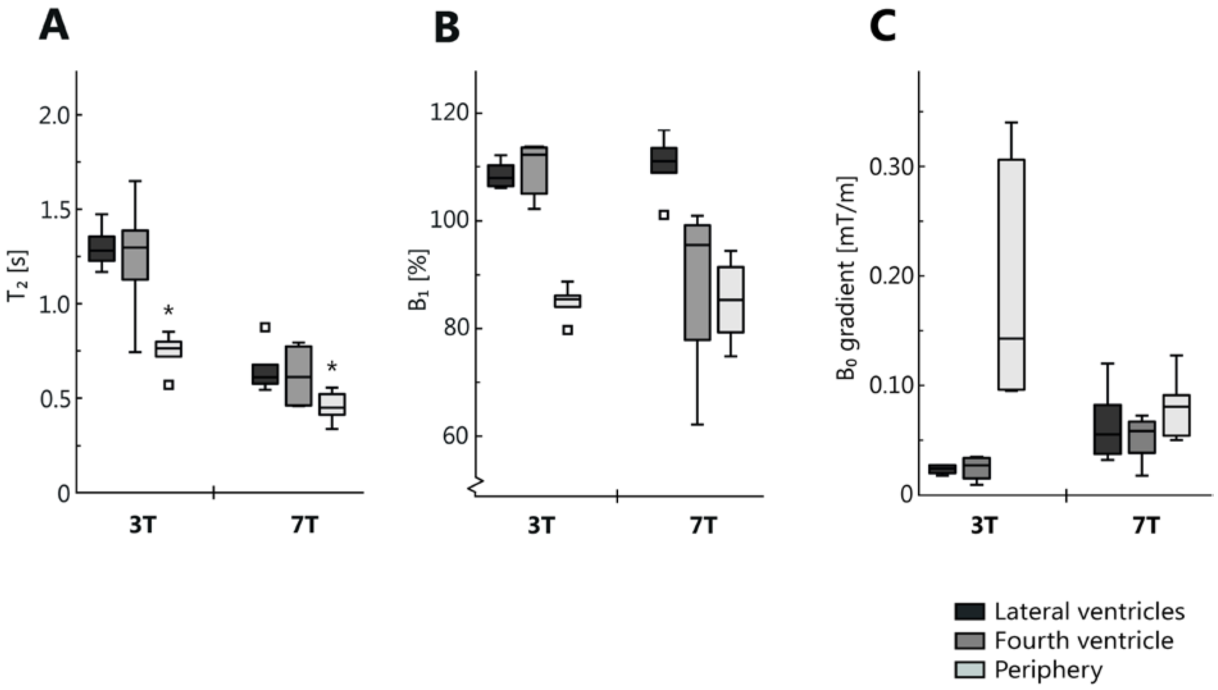


**Figure S3** In vivo results: $T_2$ (A), $B_1$ (B), and $B_0$ gradient (C) values for the three different ROIs. Outliers are represented by a square symbol. Significant differences (indicated with the grey asterisk symbol) were found between the periphery and the lateral ventricles for the SE-EPI sequence, at both field strengths (indicated using the asterisk symbol)

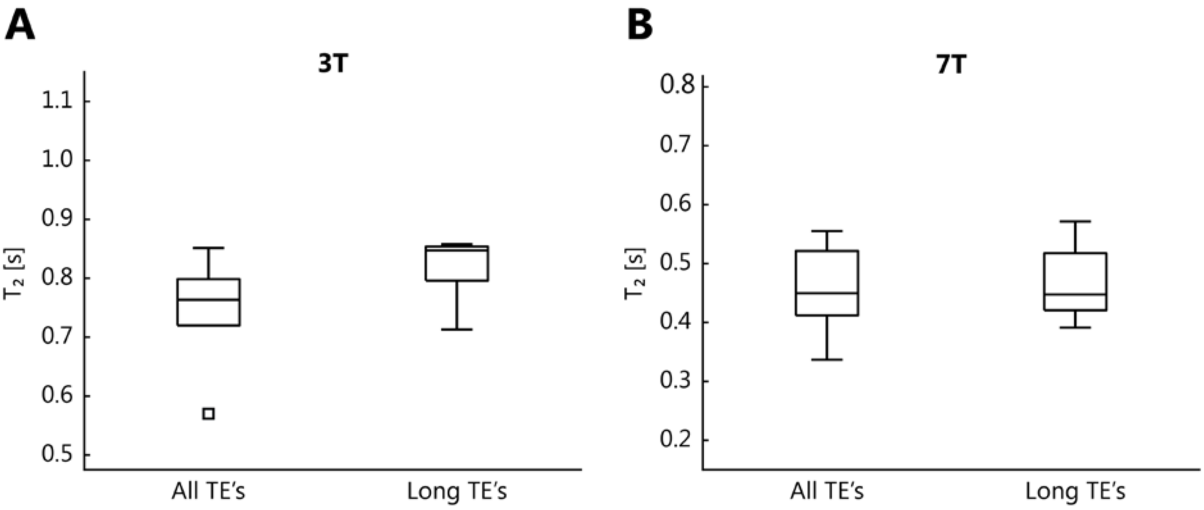


**Figure S4** $T_2$ values of peripheral CSF, resulting from the use of only the longest TEs, compared to the original analysis. Outliers are represented by a square symbol. For the SE-EPI sequence the $T_2$ increased only at 3T, but this was not significant

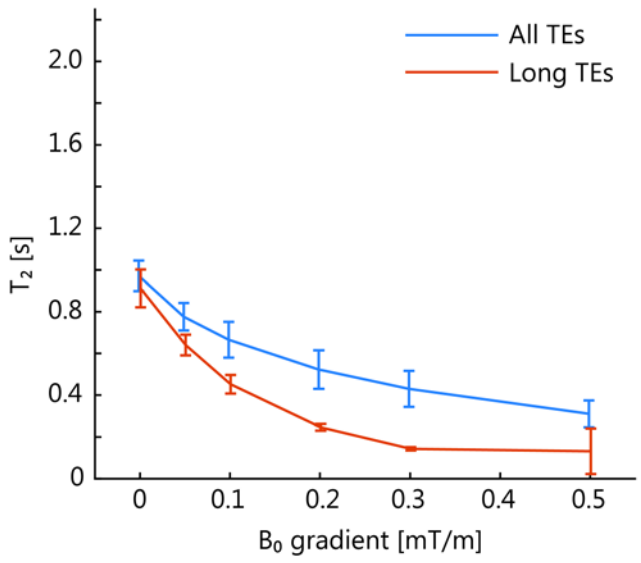


**Figure S5** $T_2$ values of the phantom, resulting from the use of only the longest TEs (orange), compared to the original analysis (blue). For the SE-EPI sequence the $T_2$ values were shorter for the analysis using only the longest TEs. The difference increased for an increasing $B_0$ gradient
